# Unveiling the oncogenic role of lncRNA PIG13-DT in hepatocellular carcinoma progression

**DOI:** 10.1080/15384047.2025.2567797

**Published:** 2025-10-09

**Authors:** Hongjie Cai, Song Chen, Shuangyan Tang, Feng Shi, Dan Zeng, Zhiqiang Wu, Fan Wang, Shuqin Huang, Dongbing Li, Wenbo Guo

**Affiliations:** a Department of Interventional Radiology, The First Affiliated Hospital of Sun Yat-sen University, Guangzhou, People's Republic of China; b Department of Minimally Invasive Interventional Therapy, State Key Laboratory of Oncology in South China, Guangdong Provincial Clinical Research Center for Cancer, Sun Yat-sen University Cancer Center, Guangzhou, People's Republic of China; c Department of Radiology, The Eighth Affiliated Hospital of Sun Yat-sen University, Shenzhen, People's Republic of China; d Department of Interventional Radiology, Guangdong Provincial People's Hospital, Guangdong Academy of Medical Sciences, Southern Medical University, Guangzhou, People's Republic of China; e Department of Medical Ultrasonics, The First Affiliated Hospital of Sun Yat-sen University, Guangzhou, People's Republic of China; f Department of Radiology, Huazhong University of Science and Technology Union Shenzhen Hospital, Shenzhen, People's Republic of China; g Molecular Genetics Laboratory, Advanced Molecular Pathology Institute of Soochow University and SANO, Suzhou, People's Republic of China

**Keywords:** Hepatocellular carcinoma, lncRNA PIG13-DT, progression, RNA-binding protein, YBX3, USP15

## Abstract

**Background:**

Hepatocellular carcinoma (HCC) is a leading cause of cancer-related mortality due to delayed diagnosis and poor prognosis. Long non-coding RNAs (lncRNAs) are key cancer regulators, yet the role of C1orf21-DT (PIG13-DT) in HCC remains unclear.

**Methods:**

We evaluated PIG13-DT expression in HCC and paired adjacent non-tumorous tissues. Functional studies were conducted using cell culture, cell-derived xenotransplantation (CDX) models, and molecular techniques including RNA pull-down, mass spectrometry, RIP-qPCR, and RNA sequencing. We explored the interplay between PIG13-DT, RNA-binding protein YBX3, and USP15 mRNA.

**Results:**

PIG13-DT was highly expressed in HCC tissues compared with normal tissues and associated with poor prognosis. Functionally, PIG13-DT enhanced cancer stem cell (CSC) function, reduced reactive oxygen species (ROS) levels, and promoted HCC cell proliferation and migration. Mechanistically, PIG13-DT interacted with YBX3, stabilizing YBX3 and promoting USP15 mRNA translation and stability, thus driving HCC progression. Clinical data from lenvatinib-treated HCC patients showed that PIG13-DT expression was correlated with poor treatment response.

**Conclusion:**

Our study identifies a novel PIG13-DT/YBX3/USP15 axis driving HCC progression, suggesting PIG13-DT as a potential biomarker and therapeutic target. This work provides new insights into HCC molecular mechanisms and offers potential diagnostic and therapeutic implications.

## 1. Introduction

Hepatocellular carcinoma (HCC) ranks as the sixth-most prevalent cancer and the third-highest cause of cancer-related deaths globally, with its incidence on the rise.^1^ Even with initiatives aimed at early screening for those at risk, more than 80% of patients are identified at an advanced stage where the disease is inoperable, resulting in significant unmet medical needs. In recent years, notable advancements have been achieved in the diagnosis and treatment of HCC. Nevertheless, individuals with unresectable HCC confront a bleak outlook. The underlying mechanisms of HCC development have been increasingly elucidated, and substantial strides have been made in understanding cancer heterogeneity.

Non-coding RNAs (ncRNAs) are genomic transcripts that do not encode proteins. Specifically, transcripts longer than 200 nucleotides are defined as long non-coding RNAs (lncRNAs).^2^ A growing body of research has highlighted the critical roles of lncRNAs in cancer initiation and progression. For instance, lncRNAs like HOTAIR, PCAT1, and PVT1 are implicated in lung tumorigenesis. Additionally, numerous studies have underscored the significant involvement of lncRNAs in HCC.^3^ The role of C1orf21 DT (PIG13-DT) in tumors is currently unclear.

The Y-box-binding protein 3 (YBX3), also known as YB−3, CSDA, DBPA, or ZONAB protein, plays a crucial role in the occurrence and development of liver diseases by binding to RNA.^4-10^ YBX3 is strongly associated with the development and progression of hepatitis B virus (HBV)-related HCC and may serve as a potential biomarker for poor prognosis.^4^^,^^5^ Further evidence has confirmed the negative correlation between dysregulated YBX3 and overall survival (OS) in HCC patients.^6^ Moreover, YBX3 has been demonstrated to be a key mediator in HCC immune evasion, hepatic ischemia-reperfusion injury, and aberrant differentiation of hepatic stem cells.^7-9^ These studies collectively suggest that YBX3 serves an important role in liver diseases, particularly in HCC, but the molecular mechanisms of YBX3 involved in the tumorigenesis and progression of HCC remain to be fully elucidated.

USP15 belongs to the ubiquitin-specific-processing (USP) protease family, which regulates a wide range of cellular processes by deubiquitinating key target proteins to modulate protein-protein interactions, trafficking, stabilization, and enzymatic activities.^11-14^ USP15 has been implicated in interactions with several proteins. For example, USP15 can deubiquitinate and stabilize the murine double minute 2 (MDM2) protein to hinder cancer cell survival,^15^ and it can deubiquitinate and suppress the activation of the ten-eleven translocation 2 (TET2) protein, thereby diminishing tumor immunogenicity.^16^ Additionally, USP15 can interact with sequestosome−1 (SQSTM1) to abolish the activation of ring finger protein 26 (RNF26), facilitating the release of diverse vesicles for fast transport and utilization of intracellular substances.^17^ A recent study has indicated an elevated expression of USP15 in HCC tumor tissues, revealing a close association with metastasis and poor prognosis.^18^ Thus, gaining a thorough understanding of this crucial modification implicated in the regulation of HCC progression could offer novel perspectives for cancer therapeutics.

In this research, we focused on examining the expression and functional role of lncRNA PIG13-DT in the progression of HCC and elucidating the associated molecular mechanisms. Our results uncover a new PIG13-DT/YBX3/USP15 axis and shed light on how PIG13-DT regulates the malignant progression of HCC, offering potential diagnostic and therapeutic targets for HCC.

## 2. Results

### 2.1. LncRNA PIG13-DT is upregulated in HCC tumor tissues and correlated with poor prognosis

**Figure 1.**
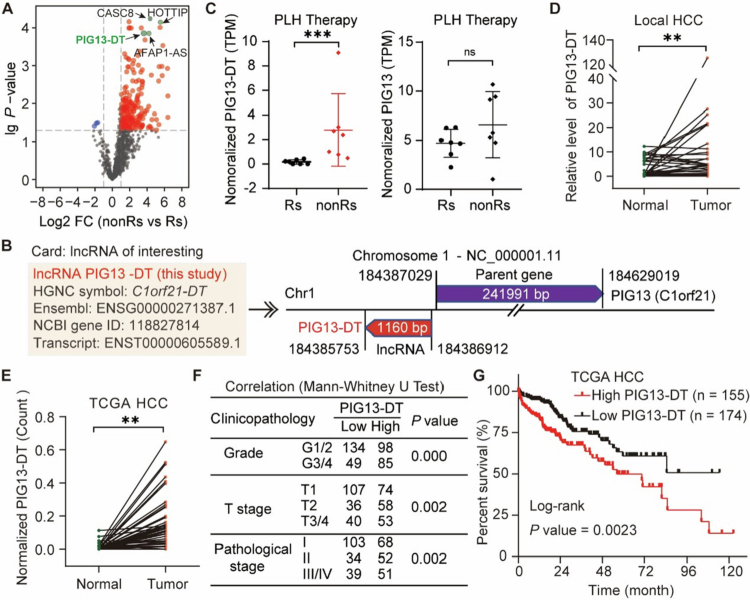
Characterization of lncRNA PIG13-DT as a novel tumor promotor in HCC. (A) Volcano plot showing significantly differentially expressed lncRNAs in RNA-seq data from paired tumor tissues of seven HCC patients. GP represents patients with good prognosis, and PP represents patients with poor prognosis. FC, fold change. (B) Information on lncRNA PIG13-DT, which is located on the reverse sequence of the promoter of the C1orf21 gene (PIG13) on chromosome 1. (C) Relative TPM (transcripts per million) of PIG13-DT and its parent gene, PIG13, in patients with good versus poor prognosis. (D) Expression of PIG13-DT in 30 pairs of HCC tissues and adjacent normal tissues from HCC patients. (E) Expression of PIG13-DT in 50 pairs of HCC tissues and adjacent normal tissues from the TCGA database. (F) Associations between PIG13-DT expression and clinicopathological features. (G) Kaplan-Meier survival analysis of overall survival in patients with high and low expression of PIG13-DT. **P < 0.01, ***P < 0.001.

To identify the potential lncRNA responsible for tumor progression in HCC, we analyzed the lncRNA transcriptional profile of seven paired HCC patients who were grouped into good and poor prognosis respectively, according to the overall survival (OS) (Table S1). Our results indicated that more lncRNAs were highly expressed in patients with poor prognosis (Figure 1A), suggesting more lncRNAs involved in these patients. Among these upregulated lncRNAs, PIG13-DT exhibited eight-fold higher expression in patients with poor prognosis than in those with good prognosis (Figure 1C). Moreover, we observed a positive correlation between PIG13-DT and its host gene PIG13 (Figure S1), whereas no significant difference in PIG13 was observed in the local cohort (Figure 1C).

To determine the full-length sequence of the PIG13-DT transcript, rapid amplification of cDNA ends (RACE)-PCR and sequencing techniques were employed. Our findings revealed that PIG13-DT is a lncRNA formed by 1160 nucleotides with a poly-A sequence tail (Figure 1B, Figure S2 and Table S2). In the local cohort, RT-qPCR analysis revealed that the expression of PIG13-DT was significantly elevated in HCC tissues relative to adjacent normal tissues (Figure 1D). Simultaneously, we examined PIG13-DT expression in the TCGA database, which includes 50 tumor samples along with both unpaired and paired peritumor tissue samples. Our findings indicated that PIG13-DT expression was also markedly upregulated in tumor tissues (Figure 1E and Figure S3), aligning with the observations in the local cohort.

To further clarify the role of PIG13-DT on HCC progression, we conducted a correlation analysis in the TCGA database. Our results demonstrated a significant positive correlation between PIG13-DT levels and tumor grade, TNM stage, and clinicopathological staging (Figure 1F). Kaplan-Meier analysis revealed that patients with high PIG13-DT expression (*n* = 155, normalized counts > 0.09) had shorter OS compared to those with low expression (*n* = 174, normalized counts < 0.09) (Figure 1G). Taken together, these findings indicated that elevated PIG13-DT expression might be responsible for poor prognosis in HCC patients.

### 2.2. The overexpression of lncRNA PIG13-DT promotes CSC function and suppresses ROS production and apoptosis in HCC cells

Cancer stem cell (CSC) are major contributors to the tumor recurrence, even after curative therapy.^19^ Moreover, hypoxia and apoptosis tolerance are both hallmark features of HCC. Thus, it is critical to investigate the potential involvement of PIG13-DT in CSC function, ROS, and apoptosis in HCC. For establishing appropriate cell models, we detected the relative expression of PIG 13-DT in four HCC cell lines (Hep3B, Huh7, SK-HEP-1, and HepG2), and found that PIG13-DT exhibited relatively lower expression in all four cell lines (Figure S4). Considering the diverse genetic and etiological background, Hep3B and Huh7 cell lines were selected for further research.

**Figure 2.**
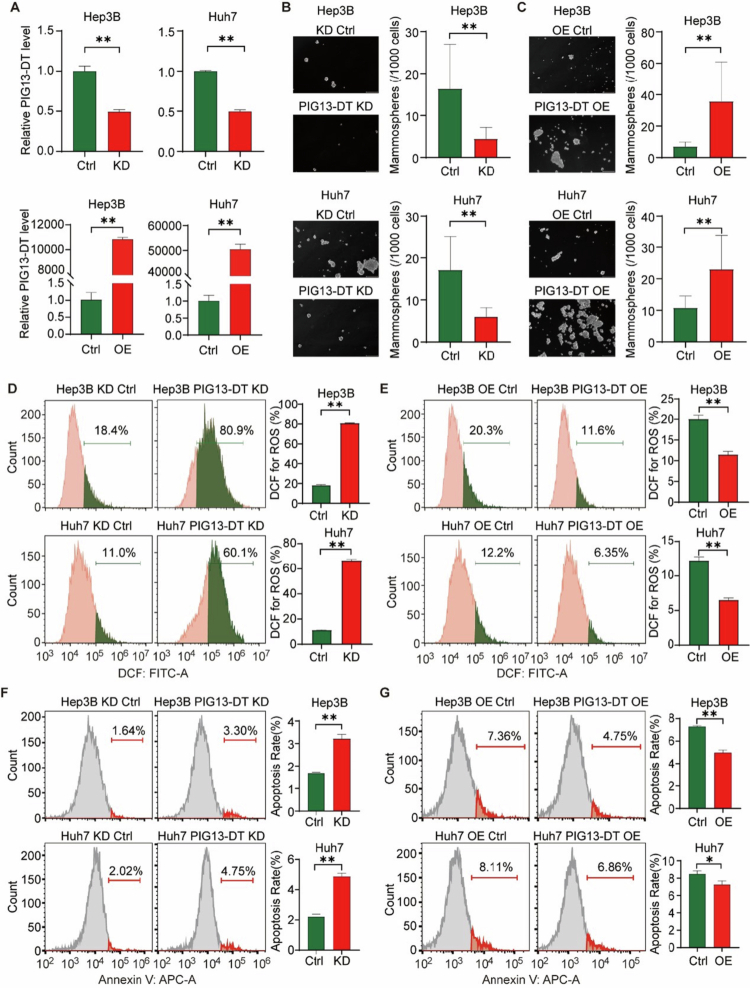
LncRNA PIG13-DT promotes CSC function and suppresses ROS production and apoptosis in HCC cells. (A) Expression of PIG13-DT in Hep3B-KD, Hep3B-OE, Huh7-KD, and Huh7-OE cell lines. (B) CSC function detected in Hep3B-KD, Hep3B-OE, Huh7-KD, and Huh7-OE cell lines using sphere-forming assay. (C-D) ROS level detected in Hep3B-KD, Hep3B-OE, Huh7-KD, and Huh7-OE cell lines using DCF-H2 staining. (E–F) Apoptosis level detected in Hep3B-KD, Hep3B-OE, Huh7-KD, and Huh7-OE cell lines by double staining with Annexin V-PI via flow cytometry. Bar graphs on the right panel were the statistical analysis of the corresponding results. KD, Knockdown of PIG13-DT. OE, Overexpression of PIG13-DT. *P < 0.05, **P < 0.01.

To investigate the biological effects of PIG13-DT, we employed shRNA vectors to achieve stable knockdown (KD) and overexpression (OE) of PIG13-DT in Hep3B and Huh7 cells. The efficiency of KD and OE was confirmed by RT-qPCR (Figure 2A). Our findings from sphere formation assay indicated that PIG13-DT KD significantly suppressed sphere formation efficiency (Figure 2B), while this effect was largely reversed by PIG13-DT OE (Figure 2C). In addition, apoptosis and ROS level were evaluated by flow cytometry. The results revealed that PIG13-DT KD significantly increased the level of ROS and promoted apoptosis in HCC cells (Figure 2D–F), while PIG13-DT OE led to the opposite trends (Figure 2E–G). The aforementioned findings suggest that PIG13-DT may significantly contribute to enhancing CSC function and inhibiting ROS production and apoptosis in HCC.

### 2.3. The overexpression of lncRNA PIG13-DT promotes proliferation, migration and invasion ability in HCC cells

**Figure 3.**
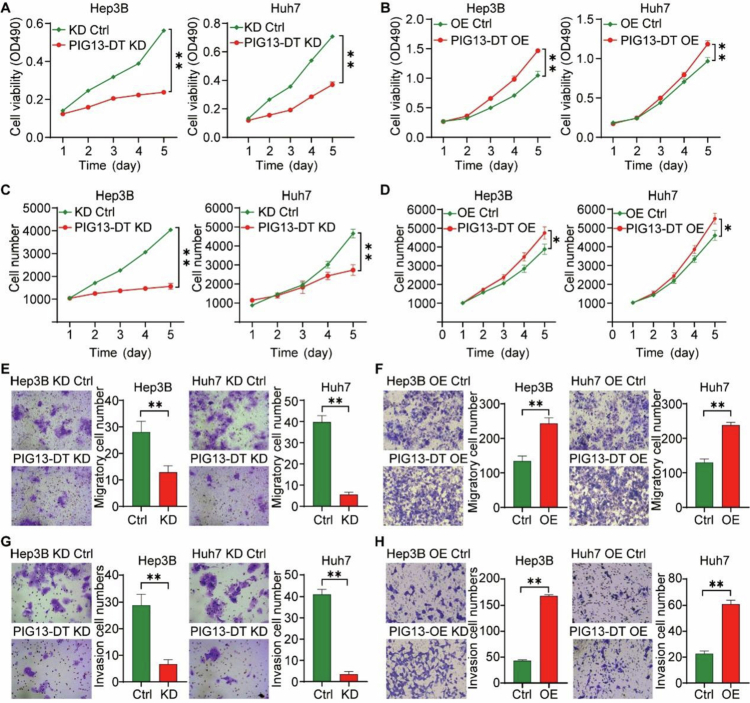
LncRNA PIG13-DT promotes proliferation, migration and invasion of HCC cells. (A–B) Cell viability of Hep3B and huh7 cells with PIG13-DT KD (A) or OE (B) determined by MTT assay for 5 d. (C–D) Cell proliferation number of Hep3B and Huh7 cells with PIG13-DT KD (C) or OE (D) determined by cellomics detection for 5 d. (E–F) Cell migration of Hep3B and Huh7 cells using transwell migration assay. (G–H) Cell invasion of Hep3B and Huh7 cells using transwell invasion assay. Bar graphs on the right panel show statistical analysis of the corresponding results. KD, Knockdown of PIG13-DT. OE, Overexpression of PIG13-DT. *P < 0.05, **P < 0.01.

Proliferation and metastasis are hallmarks of malignancies,^20^^,^^21^ driving the progression of HCC. Functional assays assessing cell viability, proliferation, migration, and invasion were performed on PIG13-DT KD and overexpression (OE) cells (Hep3B and Huh7). The MTT assay results indicated that cell number and viability were significantly reduced in PIG13-DT KD cells (Figure 3A and C), while PIG13-DT OE reversed this effect (Figure 3B and D). Similarly, the transwell migration and invasion assays showed that both migration and invasion capacities of PIG13-DT KD Hep3B and Huh7 cells were significantly reduced compared to the control group (Figure 3E and G), whereas a significant enhancement was observed in PIG13-DT OE cells (Figure 3F and H). Collectively, these findings demonstrate that PIG13-DT regulates the proliferation, migration, and invasion of HCC cells.

### 2.4. LncRNA PIG13-DT promoted HCC progression via directly binding and stabilizing YBX3 protein

**Figure 4.**
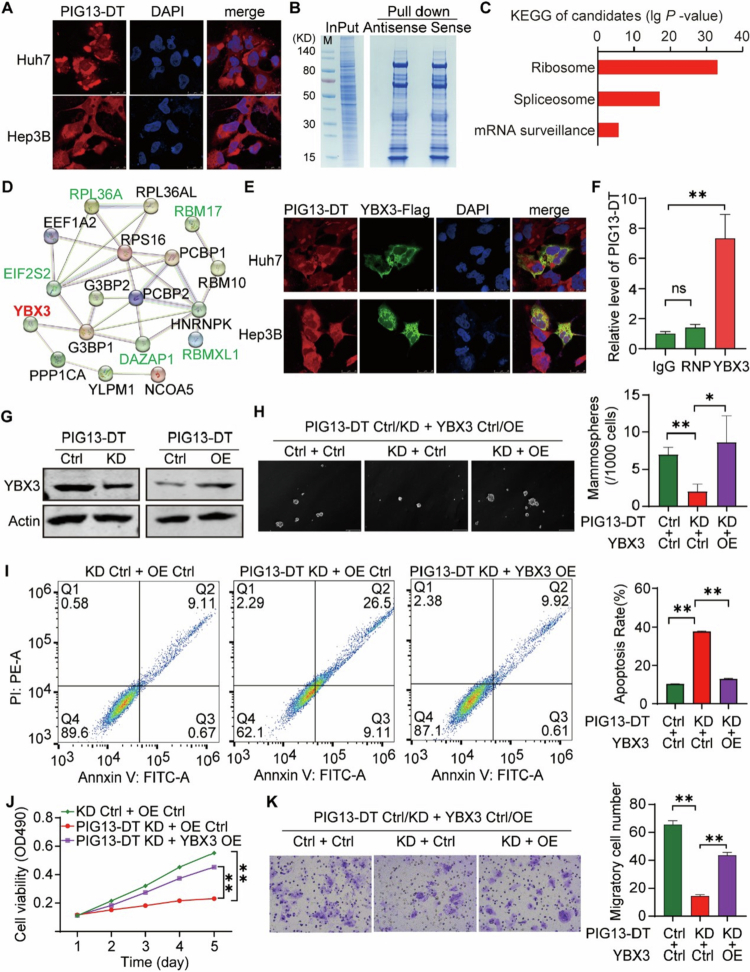
LncRNA PIG3-DT interacts directly with YBX3 protein to promote HCC progression. (A) Subcellular localization of PIG3-DT detected by FISH with Cy3-labeled RNA probe. (B) RNA pull-down assay performed by biotin-labeled PIG3-DT sense RNA and antisense RNA. (C) KEGG analysis of potential interacting proteins of PIG3-DT. (D) PPI analysis of the top 20 candidates. (E) Subcellular co-localization of PIG3-DT and YBX3-FLAG detected by FISH with Cy3-labeled RNA probe and anti-flag antibody. (F) RIP followed by RT-qPCR assay with YBX3 antibody and snRNP70 antibody, the latter as negative control. (G) Western blot of YBX3 protein in Hep3B-KD and Hep3B-OE cell lines. (H) CSC functional rescue of Hep3B using sphere formation assay. (I) Apoptosis level rescue of Hep3B using annexin V and PI dye staining. (J) Cell viability rescue of Hep3B using MTT assay. (K) Cell migration rescue of Hep3B cells using transwell migration assay. Bar graphs in the right panels show statistical analysis of the corresponding results. KD, Knockdown. OE, Overexpression. *P < 0.05, **P < 0.01.

LncRNAs can interact with RNA-binding protein (RBP) to affect the stability of lncRNA and RBP, RNA metabolism, and subcellular localization, which play pivotal roles in regulating cancer biology.^22^^,^^23^ The subcellular localization of PIG13-DT, as determined by FISH, was predominantly found in the cytoplasm (Figure 4A). Subsequently, an RNA pull-down assay and mass spectrometry were conducted to investigate the PIG13-DT complex (Figure 4B). A total of 228 potential interacting proteins were discovered. KEGG pathway revealed that the ribosome, spliceosome, and mRNA surveillance pathways were the most significantly upregulated (Figure 4C), indicating that PIG13-DT might play a vital role in regulating protein translation and RNA metabolism. Furthermore, a protein-protein interaction (PPI) network analysis of the top 20 candidates discovered that the key RBPs, including YBX3 protein, formed clusters of the interaction networks (Figure 4D). These RBPs displayed a relatively high signal-to-noise ratio (SNR) (Table S3). Due to the fact that YBX3 protein is important in global RNA metabolism and protein translation,^24^^,^^25^ we hypothesized that YBX3 could potentially interact directly with PIG13-DT. To further confirm, we performed an immunofluorescence assay and RNA immunoprecipitation (RIP)-qPCR analysis, demonstrating the co-localization of PIG13-DT and YBX3 in the cytoplasm of HCC cells (Figure 4E). Moreover, we successfully pulled down PIG13-DT RNA using YBX3 antibody-based RIP and RT-qPCR (Figure 4F). These data confirmed direct binding of YBX3 by PIG13-DT.

To assess the effect of PIG13-DT on the stability of YBX3, Western blot results demonstrated a significantly decreased level of YBX3 in the PIG13-DT KD cells, but the effect was reversed in the PIG13-DT OE cells (Figure 4G), demonstrating that the PIG13-DT/YBX3 complex stabilizes YBX3. To further validate that PIG13-DT promoted HCC progression via binding YBX3 protein, several functional rescue experiments were subsequently performed. We found that sphere formation efficiency (Figure 4H), apoptosis (Figure 4I), proliferation (Figure 4J and Figure S5), and migration abilities of HCC cells (Figure 4M) were rescued in the PIG13-DT KD and YBX3 OE groups, as compared to the PIG13-DT KD and vector control groups. Taken together, these results validated that PIG13-DT promoted HCC progression via directly interacting with YBX3, contributing to stabilize and enhance its function.

### 2.5. YBX3 protein interacts with USP15 mRNA to enhance its translation

**Figure 5.**
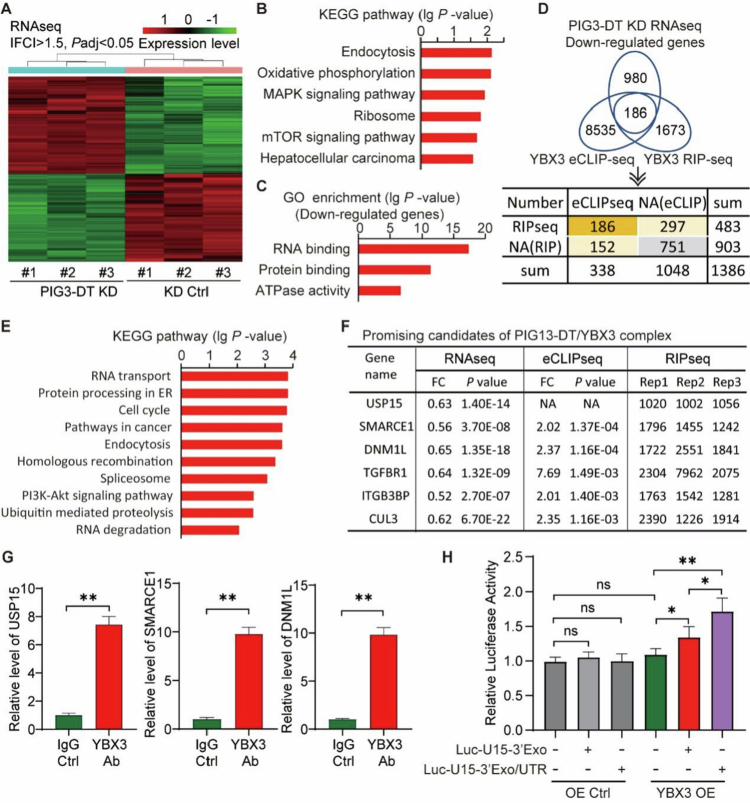
YBX3 protein binds USP15 mRNA and promotes its translation. (A) Heatmap of differentially expressed genes using RNA sequencing in Hep3B cells. (B) KEGG analysis of differentially expressed genes. (C) GO analysis of differentially expressed genes. (D) Integration of three database sets (YBX3 eCLIP-seq data, YBX3 RIP-seq data, and RNA-seq data) to identify mRNA target of the PIG13-DT/YBX3 complex. (E) KEGG analysis of 635 common genes. (F) Promising candidates of the PIG13-DT/YBX3 complex. (G) RIP and RT-qPCR performed to detect the enrichment level of target mRNAs using IP with YBX3 antibody. (H) Dual-luciferase reporter assay evaluating YBX3-mediated translational enhancement of the USP15 3' UTR. *P < 0.05, **P < 0.01.

Given the involvement of YBX3 in the regulation of RNA translation and metabolism,^24^^,^^25^ we identified the downstream targets by RNA-seq analysis (Figure 5A). The results identified 4048 differentially expressed genes between PIG13-DT KD cells and the control group, and KEGG analysis revealed that PIG13-DT could regulate both the AKT/mTOR and ERK/MAPK pathways, as well as participate in the endocytosis and oxidative phosphorylation processes (Figure 5B). In addition, GO analysis of these down-regulated genes showed that RNA binding function downregulated most significantly (Figure 5C).

Next, to further screen mRNA candidates regulated by PIG13-DT/YBX3 complex, a comprehensive approach by integrating YBX3 RIP-seq data, YBX3 eCLIP-seq data,^25^ and PIG13-DT KD RNA-seq data was utilized (Figure 5D). We found 635 mRNAs which may be regulated by both lncRNA PIG13-DT and YBX3 protein. The KEGG pathway analysis of the mRNA set revealed that these mRNAs were associated with multiple signaling pathways (Figure 5E), suggesting the crucial role of PIG13-DT/YBX3 complex in systemic RNA metabolism.

Finally, we selected six promising candidate genes, including USP15, SMARCE1, DNM1L, TGFBR1, ITGB3BP, and CUL1 (Figure 5F), which were closely relevant with HCC progression. In addition, RIP and RT-qPCR demonstrated that endogenous mRNA of USP15, SMARCE1, and DNM1L genes were successfully enriched by YBX3-specific antibodies in HCC cells (Figure 5G), further verifying the interaction between YBX3 protein and USP15, SMARCE1, and DNM1L mRNAs. Eventually, USP15 mRNA was chosen, due to its involvement in tumorigenesis and tumor progression in previous reports. Based on YBX3 eCLIP-seq data,^25^ YBX3 may directly bind 3'UTR sequences of USP15, which contained nucleotide of partial 22nd exon and 3'UTR region (Table S4). The subsequent dual-luciferase assay demonstrated that the luciferase activity of the chimeric luciferase gene was augmented by the YBX3 binding site sequences (Figure 5H), further suggesting that YBX3 can bind to USP15 mRNA to enhance translation. Taken together, these results demonstrated that YBX3 protein could interact with USP15 mRNA to enhance translation, playing a critical role in HCC progression.

### 2.6. LncRNA PIG13-DT promotes HCC progression via stabilizing USP15 mRNA

**Figure 6.**
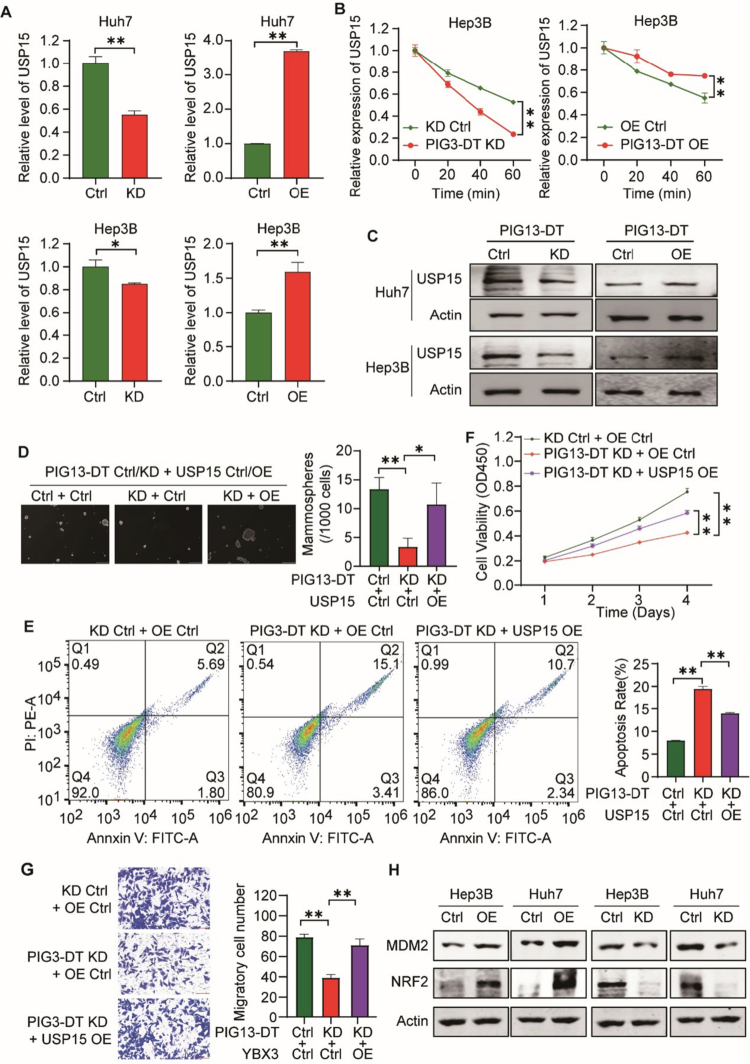
LncRNA PIG13-DT promotes HCC progression through regulating the USP15 mRNA. (A) Expression of USP15 mRNA after PIG13-DT KD or OE in HCC cells. (B) USP15 mRNA stability following actinomycin D (ACT D) treatment in HCC cells. (C) Expression of USP15 protein after PIG13-DT KD or OE in HCC cells. (D) CSC functional rescue of Hep3B cells by sphere formation assay. (E) Apoptosis level rescue of Hep3B by annexin V and PI dye staining. (F) Cell viability rescue of Hep3B cells by MTT assay. (G) Cell migration rescue of Hep3B by transwell migration assay. (H) Western blot analysis of MDM2 and NRF2, downstream effectors of the USP15 pathway. Bar graphs on the right panel show statistical analysis of the corresponding results. KD, Knockdown. OE, Overexpression. *P < 0.05, **P < 0.01.

USP15 was selected for functional follow-up based on three converging criteria: (i) its well-documented pro-tumorigenic role in HCC and other malignancies,^18^ (ii) its significant down-regulation in our PIG13-DT knock-down RNA-seq data (Figure 5A), and (iii) its direct binding to YBX3 as confirmed by independent RIP-qPCR and eCLIP-seq analyzes (Figure 5G–H). While SMARCE1 and DNM1L also exhibited YBX3-dependent enrichment, their reported functions in HCC are either context-dependent or less well characterized, and their expression changes in our RNA-seq data were more modest (fold-change < 1.5). Consequently, we prioritized USP15 as the most relevant downstream effector of the PIG13-DT/YBX3 axis for the current study. To explore whether PIG13-DT promotes HCC progression via the PIG13-DT/YBX3/USP15 axis, an actinomycin D chase assay was performed to assess the effect of PIG13-DT on USP15 mRNA stability. The findings indicated that PIG13-DT KD resulted in a significant reduction in USP15 mRNA expression, which was reversed upon PIG13-DT OE in Hep3B and Huh7 cells (Figure 6A). USP15 mRNA stability was also reduced in PIG13-DT KD cells, while it was reversed in PIG13-DT OE cells (Figure 6B). In addition, the USP15 protein was regulated by PIG13-DT in HCC cells as well (Figure 6C). These results demonstrated that PIG13-DT could regulate the USP15 mRNA expression and stability in HCC cells.

Moreover, functional rescue experiments, encompassing sphere formation, apoptosis, cell proliferation, and transwell migration assays, were carried out to confirm the impact of the PIG13-DT/YBX3/USP15 axis on HCC progression. Our results indicated that sphere formation efficiency (Figure 6D), apoptosis (Figure 6E), cell proliferation (Figure 6F), and migration (Figure 6G) were restored in the USP15 OE group compared to the OE control group. Additionally, Western blot analysis revealed that PIG13-DT could also upregulate the expression of USP15 signaling-related proteins, such as NRF2 and MDM2 (Figure 6H). Therefore, these results demonstrated that PIG13-DT promoted HCC progression via upregulating and stabilizing USP15 mRNA, and highlighted the critical role of PIG13-DT/YBX3/USP15 axis on HCC progression.

### 2.7. PIG13-DT/YBX3/USP15 axis contributes to HCC progression *in vivo*

**Figure 7.**
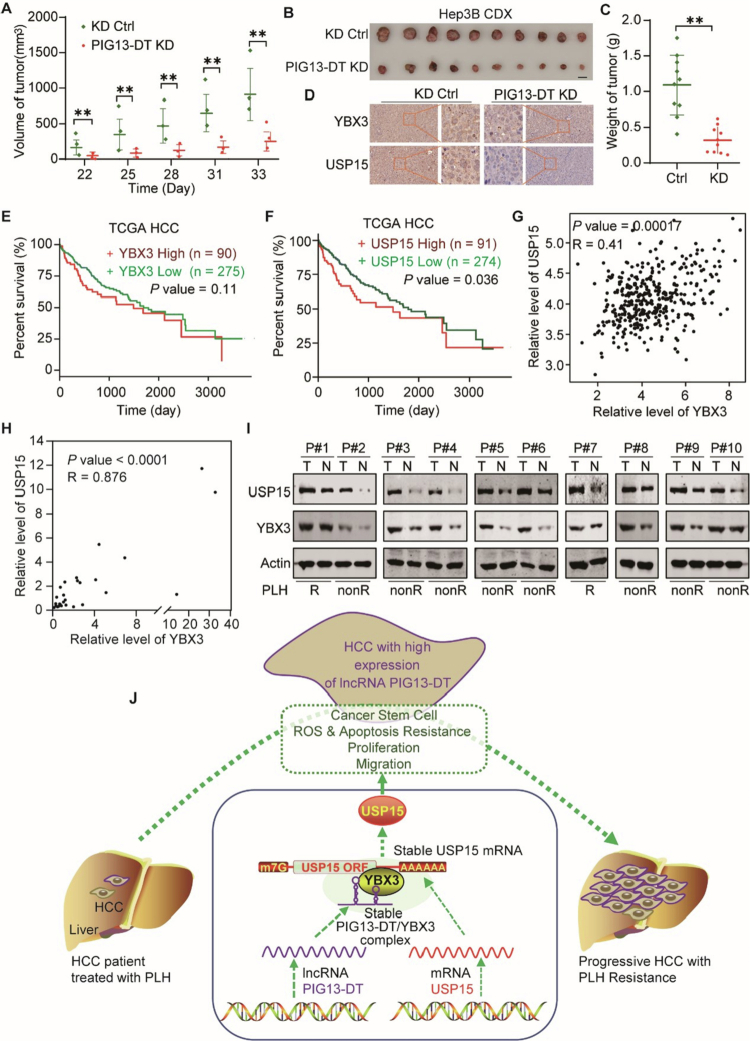
PIG13-DT/YBX3/USP15 axis contributes to HCC progression. (A-B) Growth of tumor from Hep3B cell-derived xenotransplantation (CDX) models. (C) Quantification of tumor volume and weight. (D) Immunohistochemistry of YBX3 and USP15 proteins. (E-F) Association between YBX3 expression and overall survival in HCC patients. (G) Positive correlation between PIG13-DT and YBX3 in HCC samples from the TCGA database. (H) Positive correlation between YBX3 and USP15 in HCC samples from local cohort. (I) Western blot of YBX3 and USP15 in HCC patients treated with lenvatinib and exhibiting upregulated expression of PIG13-DT. (J) Schematic model of the proposed PIG13-DT/YBX3/USP15 axis in HCC progression. LncRNA PIG13-DT promotes the malignant progression of HCC by interacting with YBX3 protein to enhance USP15 mRNA stability. **P < 0.01.

Subsequently, we established a subcutaneous xenograft tumor model in BALB/c athymic nude mice to verify the in vivo biological function of lncRNA PIG13-DT. Hep3B cells were stably transfected with vectors containing either the negative control or PIG13-DT KD. These transformed cells (negative control and PIG13-DT KD Hep3B cells) were then subcutaneously injected into the mice. The weight and volume of xenograft tumors derived from PIG13-DT KD Hep3B cells were significantly reduced compared to those from the negative control cells (Figure 7A–C & Figure S6). Immunohistochemistry revealed that PIG13-DT KD decreased YBX3 and USP15 expression in the xenograft tumors (Figure 7D). This indicated that PIG13-DT KD inhibited the in vivo proliferation of the xenografted HCC cells.

Then, we explored the potential clinical implications of the PIG13-DT/YBX3/USP15 axis on HCC progression. Through prognostic correlation analysis of the TCGA database, we found that the expression levels of YBX3 and USP15 were both significantly associated with poor prognosis (Figure 7E–F), consistent with previous studies.^6^^,^^18^ In addition, we analyzed the correlation among PIG13-DT, YBX3 and USP15 in the TCGA and local HCC cohort. The results revealed a positive correlation between PIG13-DT and YBX3 (*P* = 0.026, R = 0.12, TCGA, Figure S7A; *P* = 0.0447, R = 0.39, local, Figure S7C), a positive correlation between PIG13-DT and USP15 (*P* = 0.00092, R = 0.17, TCGA, Figure S7B; *P* = 0.0261, R = 0.43, local, Figure S7D), and a strong positive correlation between YBX3 and USP15 (*P* = 0.00017, R = 0.41, TCGA, Figure 7G; *P *< 0.0001, R = 0.88, local, Figure 7H). Western blot confirmed their up-regulation in HCC tissues. The expression of PIG13-DT was measured by RT-qPCR, and we observed that the expression of lncRNA PIG13-DT in tumor tissues was significantly elevated compared to normal tissues (Figure S8). Western blot analysis revealed that the levels of YBX3 and USP15 were also higher in HCC tissues than in normal tissues (Figure 7I). Among the ten lenvatinib-treated patients, all tumors exhibited high PIG13-DT expression; however, this observation is preliminary and hypothesis-generating owing to the limited sample size (Figure 7I). These data suggest a potential link between the PIG13-DT/YBX3/USP15 axis and lenvatinib refractoriness, but the association remains preliminary and requires validation in larger, adequately powered cohorts.

## 3. Discussion

HCC is a highly heterogeneous malignancy characterized by increasing incidence and substantial mortality. Despite advances in diagnostics and therapeutics, the prognosis for patients with unresectable HCC remains poor. Elucidating the molecular mechanisms underlying HCC progression is essential for identifying new diagnostic biomarkers and therapeutic targets. In this study, we investigated the role of lncRNA PIG13-DT in HCC progression and explored its underlying mechanisms.

The molecular mechanism underlying PLH therapy in HCC may involve comprehensive, synergistic tumor suppression, including inhibition of the traditional classical signaling pathway utilizing multi-kinase inhibitors, promotion of apoptosis through ischemia/hypoxia and chemotherapy, and enhancement of immune response against tumors.^26^ Lenvatinib inhibits both FGFR4 signaling and Treg-mediated immunosuppression, thereby improving the efficacy of HCC immunotherapy.^27^ The resistance of HCC to PLH therapy may be attributed to various factors, such as the differentiation of CSC function,^19^ apoptosis resistance under hypoxic stress,^28^ abnormal activation of canonical signaling pathways in tumor cells,^29^ and tumor immune escape. In this study, we concentrated on examining several key aspects of HCC in the context of PLH therapy, including CSC function, ROS and apoptosis, proliferation, and migration. These experiments were designed to identify potential new biomarkers and therapeutic targets, and to elucidate the molecular mechanisms underlying resistance to PLH therapy.

Our research demonstrated that PIG13-DT is markedly elevated in HCC tissues relative to adjacent normal tissues, and its high expression is linked to an unfavorable prognosis in HCC patients. This indicates that PIG13-DT could serve as a biomarker for predicting the prognosis of HCC patients. The increased expression of PIG13-DT in HCC tissues and its association with poor prognosis underscore its value as a diagnostic and prognostic marker, which may facilitate early detection and risk stratification in HCC patients.

Functionally, we demonstrated that PIG13-DT overexpression enhances CSC function, suppresses ROS production and apoptosis, and promotes the proliferation, migration, and invasion of HCC cells. These results indicate that PIG13-DT plays a critical role in the malignant progression of HCC. The enhancement of CSC function by PIG13-DT may contribute to tumor recurrence and resistance to therapy, while the suppression of ROS and apoptosis may promote tumor survival and progression. The promotion of proliferation, migration, and invasion further supports the role of PIG13-DT in driving HCC aggressiveness. Although sphere-formation assays provide a robust functional correlation of CSC activity, future studies will formally quantify core stemness markers (e.g., OCT4, CD133, SOX2) to refine our understanding of how PIG13-DT modulates cellular plasticity in HCC.

LncRNAs in the cytoplasm can function as RBP partners,^22^^,^^23^ mediating signal transduction pathways, translational programs, and post-transcriptional control of gene expression.^30^ Our study showed that PIG13-DT is primarily localized in the cytoplasm of HCC cells, prompting us to investigate its interaction with RBPs. We found that PIG13-DT directly interacts with the RNA-binding protein YBX3, leading to increased stability of YBX3. This interaction is vital for the progression of HCC, given that YBX3 has been identified as a key factor in the development and progression of liver diseases, including HCC. YBX3 is implicated in numerous processes, such as differentiation of hepatic stem cells, hepatic ischemia-reperfusion injury, and immune evasion in HCC.^6-9^ Our findings uncover the pivotal role of YBX3 as a crucial RBP partner of PIG13-DT in driving HCC progression. Recent work has further highlighted the diversity of lncRNA–RBP regulatory networks in HCC, including the roles of HOTAIR–YBX1, lncRNA-HMG–YBX3 (PMID: 39225204) and MALAT1–IGF2BP1 (PMID: 32436837), underscoring the general importance of such interactions in liver-cancer progression.^31-33^

Given the critical role of the YBX3 protein in regulating translation and stability,^24^ we propose that PIG13-DT promoted HCC progression via the PIG13-DT/YBX3 complex to regulate translational programs. To identify the target mRNA of the PIG13-DT/YBX3 complex, we integrated three omics datasets and found that USP15 mRNA is a significant target of YBX3. We demonstrated that PIG13-DT contributes to HCC progression by stabilizing USP15 mRNA. USP15 has been demonstrated to facilitate tumor growth and suppress apoptosis in HCC cells, which aligns with our findings.^18^ USP15 serves as a key regulator in various cancer-related processes, including signaling pathways, apoptosis, tumor immunity, and vesicle trafficking.^11-13^ Collectively, our findings suggest that the lncRNA PIG13-DT/YBX3 protein/USP15 mRNA complex is a critical signal axis in HCC progression.

In vivo experiments utilizing a subcutaneous xenograft tumor model in BALB/c athymic nude mice further confirmed the role of PIG13-DT in HCC progression. PIG13-DT knockdown led to reduced expression of YBX3 and USP15 proteins in vivo, demonstrating the crucial role of the PIG13-DT/YBX3/USP15 axis in HCC progression. Moreover, our study showed positive correlations among PIG13-DT, YBX3, and USP15 in both the TCGA and local HCC cohorts. Notably, in a small exploratory cohort of 8 lenvatinib-treated HCC patients, tumors with elevated PIG13-DT expression appeared to exhibit a trend toward poorer response; however, this observation is preliminary and requires validation in larger, adequately powered studies before any clinical inference can be drawn.

In summary, our study identified a novel lncRNA PIG13-DT that is upregulated in HCC patients who are unresponsive to PLH therapy. We unraveled a novel PIG13-DT/YBX3/USP15 axis that contributes to the malignant progression of HCC. Functionally, lncRNA PIG13-DT promotes CSC function, suppresses ROS and apoptosis, and facilitates the proliferation and migration of HCC cells. Mechanistically, PIG13-DT interacts with YBX3 protein and increases its accumulation, which in turn promotes HCC progression by enhancing USP15 mRNA stability and protein accumulation. These findings offer valuable insights into the potential of targeting PIG13-DT as a therapeutic strategy for HCC that is resistant to PLH therapy. Future research should focus on further elucidating the detailed molecular mechanisms and exploring the therapeutic potential of targeting the PIG13-DT/YBX3/USP15 axis in HCC.

However, several limitations should be acknowledged. This study focused primarily on the PIG13-DT/YBX3/USP15 axis, but the progression of HCC involves multiple complex signaling pathways. The study did not fully explore whether PIG13-DT affects the progression of HCC through other signaling pathways or synergistic effects with other molecules. When evaluating the response to lenvatinib treatment, the analysis was based solely on the expression level of PIG13-DT, without integrating additional clinical indicators or biomarkers, potentially overlooking other factors that affect treatment response, resulting in a one-sided outcome. Although PIG13-DT is considered a potential therapeutic target, research has not explored the feasibility and safety of treatment methods targeting PIG13-DT. For example, the development and application of RNA interference technology or small molecule inhibitors require further research. Although our integrative analyzes identify SMARCE1 and DNM1L as additional YBX3-associated transcripts (Figure 5G), their functional contribution to the PIG13-DT/YBX3-mediated phenotype remains to be elucidated and will be the subject of ongoing work. Although sphere formation provides a functional readout for stem-like behavior, future studies will formally assess the expression of core stemness markers (EpCAM, SOX2, OCT4) at both the mRNA and protein levels to refine our understanding of how PIG13-DT modulates cellular plasticity in HCC. Future work will employ unbiased ubiquitin-remnant profiling to identify the full spectrum of USP15 substrates and clarify how its de-ubiquitinating activity contributes to HCC aggressiveness. While the current results provide functional evidence for each node of the PIG13-DT/YBX3/USP15 axis, future integrative gain- and loss-of-function studies targeting all three components simultaneously will further dissect their concerted role in HCC progression. Although the observed changes in YBX3 protein abundance – without concomitant alterations in its mRNA – are consistent with a stabilization mechanism, a formal cycloheximide chase experiment will be conducted in follow-up work to quantify YBX3 half-life under varying PIG13-DT levels. Although our luciferase reporter and rescue data strongly suggest that YBX3 enhances USP15 translation, formal polysome or ribosome-profiling analyzes will be pursued in follow-up studies to quantify the translational efficiency of USP15 mRNA under varying YBX3 levels. In view of the small sample size, a formal multivariable analysis adjusting for tumor stage, liver function, and additional clinical variables will be undertaken in a forthcoming prospective study designed specifically to assess PIG13-DT as a predictive biomarker for lenvatinib response.

In summary, our results establish the lncRNA PIG13-DT as a key driver of HCC progression through a newly defined PIG13-DT/YBX3/USP15 axis. Functionally, overexpression of PIG13-DT enhances CSC function, inhibits ROS production and apoptosis, and promotes the proliferation, migration, and invasion of HCC cells. Mechanistically, PIG13-DT interacts directly with the RNA-binding protein YBX3, leading to increased stability of YBX3, which in turn promotes HCC progression by enhancing USP15 mRNA stability and protein accumulation. Our findings hold significant implications for the diagnosis and treatment of HCC. The PIG13-DT/YBX3/USP15 axis represents a novel pathway that contributes to the malignant progression of HCC. The upregulation of PIG13-DT in HCC tissues and its correlation with poor prognosis suggest that it could serve as a valuable potential biomarker for predicting disease outcome. Moreover, the critical role of this axis in HCC progression highlights PIG13-DT as a promising therapeutic target. Targeting PIG13-DT may disrupt the PIG13-DT/YBX3/USP15 axis, thereby inhibiting the progression of HCC and improving patient outcomes. Future studies should focus on further elucidating the detailed molecular mechanisms by which the PIG13-DT/YBX3/USP15 axis regulates various biological processes in HCC cells. Additionally, the development of therapeutic strategies targeting PIG13-DT, such as small molecule inhibitors or RNA interference technologies, should be explored to translate these findings into clinical applications. Overall, our study provides new insights into the molecular pathogenesis of HCC and offers potential avenues for the development of novel diagnostic and therapeutic approaches for this aggressive malignancy.

## 4. Materials and methods

### 4.1. Clinical specimen collection

Human HCC paired tumor and adjacent non-tumor tissue samples were collected from patients who underwent PD−1 (PLH) treatment at the Department of General Surgery, the First Affiliated Hospital of Sun Yat-sen University. To further elucidate the role of the PIG13-DT/YBX3/USP15 axis in HCC, we obtained paired tumor and normal tissue samples from 10 HCC patients who received lenvatinib treatment at the First Affiliated Hospital of Sun Yat-sen University. Written informed consent was obtained from all participants. Additionally, clinical samples from The Cancer Genome Atlas (TCGA, https://tcga-data.nci.nih.gov/) database were downloaded for lncRNA and mRNA expression analysis.

### 4.2. Cell culture

Human HCC cell lines, including Hep3B, Huh7, SK-HEP-1, and HepG2, were sourced from the National Collection of Authenticated Cell Cultures in Shanghai, China. These cells were maintained in Dulbecco's Modified Eagle Medium (DMEM) enriched with 10% fetal bovine serum (FBS) and 1% penicillin-streptomycin, and were incubated at 37 °C in a humidified environment with 5% CO_2_.

### 4.3. Cell-derived Xenotransplantation (CDX)

This experiment was carried out in compliance with the Animal Welfare and 3Rs (Replacement, Reduction, and Refinement) guidelines and was approved by the Genechem Institutional Animal Care and Use Committee (Approval No.: GSGC0198074). A total of 20 female BALB/c nude mice (4 weeks old, average weight: 13 g) were obtained from SLAC Animal in Shanghai and were divided into two groups: the PIG13-DT knockdown (KD) group (*n* = 10) and the KD control (Ctrl) group (*n* = 10). Human Hep3B cells were micro-injected at a density of 4 × 10^6^ into the right flank limbs of these mice. Tumor sizes were measured on days 22, 25, 28, 31, and 33 post-transplantation. Tumor volume was calculated using the formula V = π/6 × L × W^2^ (where L is the major axis and W is the minor axis). On day 33, all mice were euthanized, and tumors were collected and weighed. For immunohistochemical staining, each tissue sample was immediately fixed in 4% formalin and then embedded in paraffin.

### 4.4. RACE-PCR

Total RNA was extracted using the TRIzol Plus RNA Purification Kit (Thermo Fisher Scientific). The 5ʹ RACE and 3ʹ RACE assays were performed using the GeneRacer™ Kit (Thermo Fisher Scientific). The following gene-specific primers (GSP) were utilized for PCR: 

5ʹ RACE PIG13-DT GSP1: 5ʹ-CGCCTCTACCGAATCGCGGATGCT−3ʹ,

5ʹ RACE PIG13-DT GSP2: 5ʹ-GGGTCAGTATGGAGGCGGATCGGT−3ʹ,

3ʹ RACE PIG13-DT GSP1: 5ʹ-CCCTCATTGCCTTAGACACGCGAGGAA−3ʹ,

3ʹ RACE PIG13-DT GSP2: 5ʹ-CCCCCAGAGTGAGAACACCCAAGA−3ʹ.

### 4.5. LncRNA knockdown and overexpression

The sequences of shPIG13-DT (GCGCTTCACTGCTCCCTCATT) and shRNA Ctrl (TTCTCCGAACGTGTCACGT) were designed to construct PIG13-DT knockdown lentiviruses (GCB). The full length of PIG13-DT was synthesized togenerate PIG13-DT-overexpressing lentiviruses (GCB). PIG13-DT knockdown or overexpression lentiviruses were infected into HCC cells to construct stable cell lines, which were verified by RT-qPCR technology.

### 4.6. RT-qPCR

Total RNA was isolated from HCC cells using Trizol reagent (Invitrogen). cDNA was synthesized following the manufacturer's protocol (TaKaRa), and RT-qPCR was conducted using SYBR Premix Ex Taq II (TaKaRa) on a LightCycler system (Roche).^34^ The PCR amplification conditions were as follows: initial denaturation at 94 °C for 30 s, followed by 40 cycles of amplification (94 °C for 5 s, 60 °C for 30 s, and 72 °C for 30 s). The expression levels of PIG13-DT, YBX3, and USP15 were quantified using the 2^-ΔΔCt^ method, normalized to GAPDH or Actin. Each experiment included triplicate wells.

The primers used were:

PIG13-DT forward: 5ʹ-ACCCTCACAGAAGCTCAATCA−3ʹ,

PIG13-DT reverse: 5ʹ-AAAGACATCCAAAGGCTATCAA−3ʹ;

YBX3 forward: 5ʹ-ACCGGCGTCCCTACAATTAC−3ʹ,

YBX3 reverse: 5ʹ-GGTTCTCAGTTGGTGCTTCAC−3ʹ;

USP15 forward: 5ʹ-CGACGCTGCTCAAAACCTC−3ʹ,

USP15 reverse: 5ʹ-TCCCATCTGGTATTTGTCCCAA−3ʹ;

GAPDH forward: 5ʹ-TGACTTCAACAGCGACACCCA−3ʹ,

GAPDH reverse: 5ʹ-CACCCTGTTGCTGTAGCCAAA−3ʹ;

Actin forward: 5ʹ-CACTCTTCCAGCCTTCCTT−3ʹ,

Actin reverse: 5ʹ-ACAGGTCTTTGCGGATGT−3ʹ.

### 4.7. Spheroid formation assay

Adherent tumor cells were detached using trypsinization and then resuspended in DMEM/F12 medium (Gibco) fortified with 4 μg/mL heparin (Sigma-Aldrich), B27 (Gibco), 20 ng/mL human recombinant epidermal growth factor (Peprotech), and 20 ng/mL human recombinant basic fibroblast growth factor (Peprotech). These single-cell suspensions were cultured in 6-well ultra-low attachment plates (Corning) at a density of 1000 cells per well for 10−14 d at 37 °C. The resultant spheres were visualized under a microscope and quantified using ImageJ software.

### 4.8. Reactive oxygen species (ROS) assay

HCC cells were dissociated, and 1 mL of medium containing 1 × 10^6^ cells was added to each well of a six-well plate. The cells were then cultured at 37 °C in a 5% CO_2_ incubator for 48 h. DCFH-DA (Sigma-Aldrich) was introduced into the cell suspensions at the recommended concentration. Following a 30-min incubation at 37 °C in the dark, the suspensions were washed with PBS and resuspended in 200 μL PBS. The samples were analyzed using a FACSCanto™ II flow cytometer (BD Biosciences), and the data were processed using FlowJo software (Tree Star).

### 4.9. Cell apoptosis

Cell apoptosis was evaluated using flow cytometry (FCM).^35^ Cells were collected by centrifugation at 1500 rpm for 5 min, washed with 1 × binding buffer and 1 × staining buffer, and then stained with 5 μL of Annexin V-APC and/or propidium iodide (PI) staining solution in the dark. The stained cells were then detected using FCM.

### 4.10. Cell proliferation

Hep3B and Huh7 cells infected with shPIG13-DT or shRNA control were plated in a 96-well plate at a density of 2000 cells per well in 100 μL DMEM. Cell proliferation was monitored daily for 5 d to construct cellular proliferation curves.^36^ Additionally, cell proliferation rates were assessed using the MTT assay. At specified time points, MTT solution was added to each well and incubated for 2 h. Subsequently, 150 μL of DMSO was added to dissolve the formazan crystals for 20 min, and the absorbance at 570 nm was measured.

### 4.11. Cell migration and invasion assay

Cells transfected with shPIG13-DT or shRNA control were collected, and 1 × 10^5^ cells in 200 μL serum-free DMEM were seeded onto a Transwell chamber coated with Matrigel (invasion) or left uncoated (migration). Subsequently, 800 μL DMEM containing 10% FBS was added to the lower chamber, and the cells were cultured for 24−48 h. Following this, the cells were fixed with 4% paraformaldehyde and stained with 0.1% crystal violet. The number of cells was then counted under a light microscope. Each assay was conducted three times, with triplicate wells used for each experiment.

### 4.12. FISH

The subcellular localization of PIG13-DT in HCC cells was assessed using the Ribo™ FISH Kit and a PIG13-DT probe mix labeled with Cy3® 532 nm dye (Ribo). The subcellular localization of YBX3-Flag in HCC cells was determined using an anti-Flag antibody (CST) and Donkey Anti-Rabbit IgG H&L labeled with Alexa Fluor® 488 nm dye (Abcam). HCC cells were fixed with 4% paraformaldehyde, treated with protease reagent, incubated with prehybridization buffer at 40 °C for 4 h, and then hybridized with the Cy3-labeled RNA probe mix in a humidified chamber at 40 °C overnight. After washing and blocking, the slides were sequentially incubated with the anti-Flag antibody and the Alexa Fluor-conjugated anti-Rabbit antibody. Following additional washing, the slides were incubated with SABC-FITC at 37 °C for 30 min and then examined using a Nikon inverted fluorescence microscope.

### 4.13. Pull-down and mass spectrometry detection

For RNA pull-down experiments, biotin-labeled PIG13-DT transcripts and their antisense counterparts were synthesized through in vitro transcription using T7 RNA polymerase and Biotin RNA Labeling Mix (Roche). These were then incubated with Hep3B cell lysates at 37 °C for 4 h. Streptavidin-conjugated agarose beads were employed for centrifugal enrichment of the complexes. The precipitated components were resolved by SDS-PAGE. Subsequently, proteins interacting with the lncRNA were identified via mass spectrometry and matched against the human proteomic library.

### 4.14. RIP-qPCR

RIP was performed to detect the interaction between PIG13-DT and YBX3. Briefly, the YBX3 antibody (Abcam) was used to precipitate YBX3 and co-precipitated RNAs were detected by RT-qPCR. The isotype IgG was used as a control. The primers for detecting PIG13-DT and USP15 were: 

PIG13-DT forward: 5ʹ-ACCCTCACAGAAGCTCAATCA−3ʹ,

PIG13-DT reverse: 5ʹ-AAAGACATCCAAAGGCTATCAA−3ʹ;

USP15 forward: 5ʹ-CGACGCTGCTCAAAACCTC−3ʹ,

USP15 reverse: 5ʹ-TCCCATCTGGTATTTGTCCCAA−3ʹ;

SMARCE1 forward: 5ʹ-CGACGAGAACATTCCGAT−3ʹ,

SMARCE1 reverse: 5ʹ-TCCTTCCTCTGCCATACTG−3ʹ;

DNM1L forward: 5ʹ-CAACTGGAGAGGAATGCTG−3ʹ,

DNM1L reverse: 5ʹ-CAACAGGAACTGGCACATC−3ʹ.

### 4.15. RNA sequencing analysis

Total RNA was extracted from HCC and normal tissues using TRIzol reagent (Invitrogen) and further processed with the Ribo-off rRNA Depletion Kit (Vazyme) to remove ribosomal RNA. The purified RNA samples were then used to construct RNA sequencing libraries with the VAHTS Total RNA-seq (H/M/R) Library Prep Kit for Illumina (Vazyme). The libraries were sequenced on the Illumina platform using a 150 bp paired-end configuration. The sequencing reads were aligned to the human reference genome (GRCh38) using the HISAT2 spliced read aligner. Annotations for lncRNAs and mRNAs were obtained from the GENCODE (v25) database. Additionally, lncRNA and mRNA expression data, along with corresponding clinical information from an HCC cohort comprising 371 tumor tissues and 50 normal controls, were retrieved from the TCGA database.

### 4.16. Western blot

Protein lysates were collected using RIPA buffer lysis. The protein concentration was measured using a BCA protein assay kit (Thermo). The proteins were separated on 10% SDS-PAGE gels and subsequently transferred to a polyvinylidene difluoride (PVDF) membrane. The PVDF membrane was blocked with 5% nonfat skim milk at room temperature and incubated with primary antibodies targeting YBX3 (1:2,000, Atlas), USP15 (1:1,000, Abcam), and ACTB (1:1,500; Abcam). The corresponding secondary antibody was diluted and incubated with the membrane at room temperature for 1 h. Finally, the protein expression was examined using the BioSpectrum Imaging System (UVP).

### 4.17. Actinomycin D RNA stability assay

Hep3B cells were exposed to 5 μg/mL actinomycin D (ACT D) (Sigma), and RNA was extracted using the Zymo Research Quick-RNA™ MiniPrep at various time points: 0 minutes (untreated), 20, 40, and 60 min post-treatment. The obtained RNA samples were then subjected to cDNA synthesis and RT-qPCR following the aforementioned protocol. RNA decay was assessed by normalizing RT-qPCR values relative to the baseline (0 h) for each condition. The half-life values were determined through semi-logarithmic linear regression analysis to identify the best-fit line for the data.

### 4.18. Dual-luciferase reporter assay

The specific sequence of USP15 targeted by YBX3 was selected to create the pmirGLO Dual-Luciferase Target Expression Vector (Promega) to evaluate the direct binding of the potential USP15 sequence by the YBX3 protein. The YBX3 overexpression construct and the USP15 reporter construct were co-transfected into 293 T cells. Following a 24-h transfection period, Firefly luciferase activity was measured using the Dual-Luciferase Reporter Assay System (Promega) and normalized to Renilla luciferase activity. Each experiment was conducted at least three times.

### 4.19. Statistical analysis

Data are expressed as mean ± standard deviation and were analyzed using SPSS 19.0 software. The comparison of clinical case data used the Mann–Whitney U test. Survival analysis was conducted using the Kaplan-Meier method. The correlation between PIG13-DT expression and YBX3 and USP15 was assessed using the Spearman rank correlation coefficient. Significant differences between two groups were determined using Student's *t* test, with *P-*value < 0.05 considered statistically significant. All experiments were performed at least three times independently to ensure reproducibility and reliability of the results. Each experiment included triplicate wells for each condition, and the data were pooled for statistical analysis.

## Consent for publication

Not applicable.

## Supplementary Material

Supplementary material Supplementary Figures CLEAN COPY

Supplementary material Supplementary Tables CLEAN COPY

## Data Availability

The data that support the findings of this study are available from the corresponding author upon reasonable request.
