# Supplementary material for: Unveiling the oncogenic role of lncRNA PIG13-DT in hepatocellular carcinoma progression
Source: Cancer Biol Ther. 2025 Oct 9;26(1):2567797. doi: 10.1080/15384047.2025.2567797 (PMC12520071; doi:10.1080/15384047.2025.2567797)
Supplement: Supplementary material — Supplementary Figures CLEAN COPY [file KCBT_A_2567797_SM8985.docx]

**
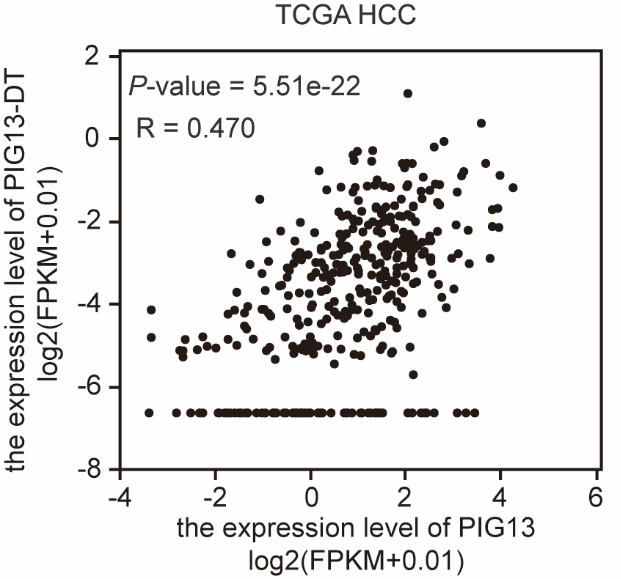
**

**Figure S1**. **Correlation between PIG13 gene and PIG13-DT gene, as well as their expression in HCC patients with PLH therapy.**


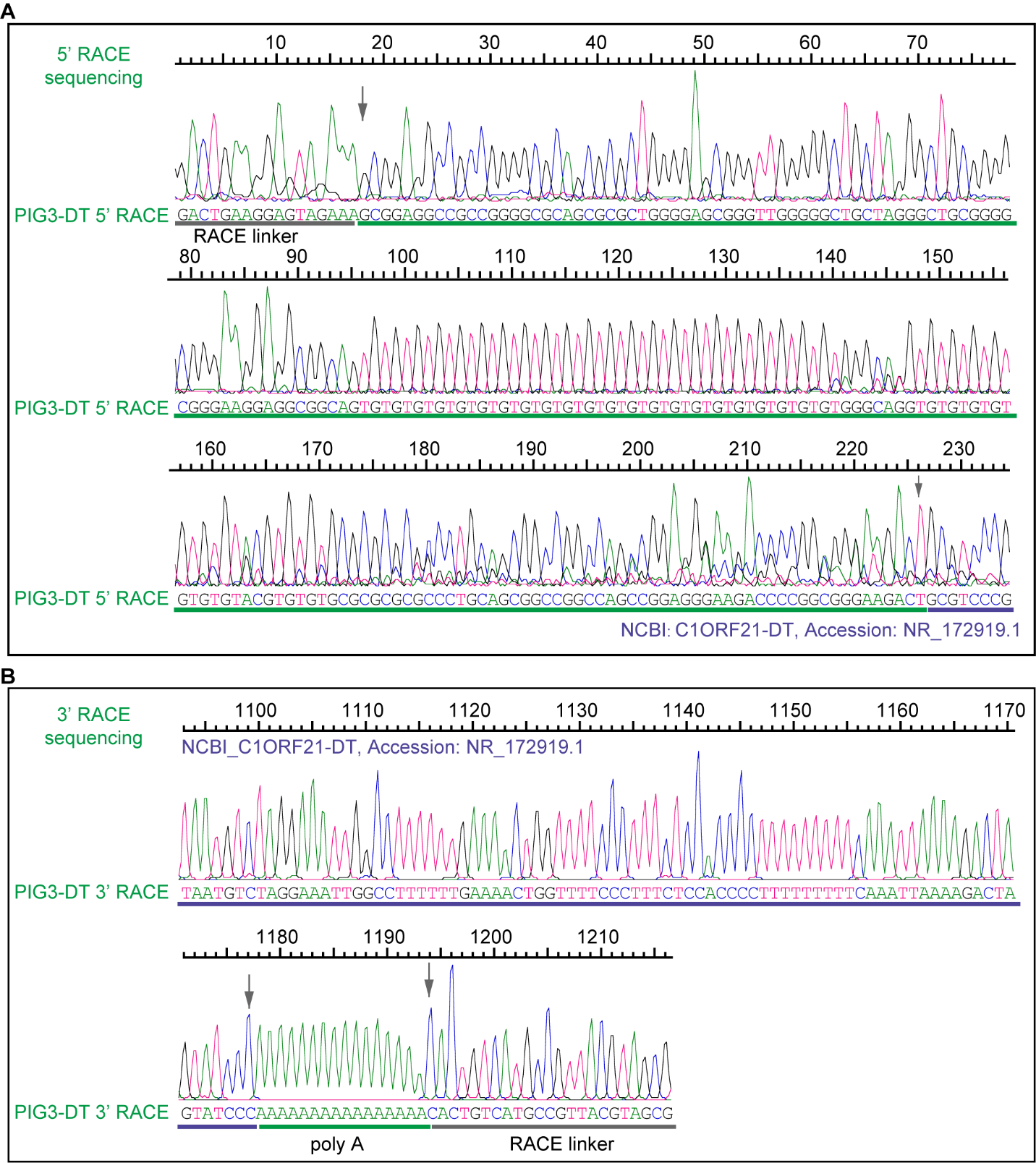


**Figure S2**. **RACE assembly of PIG13-DT using RACE sequencing.** (A) 5’RACE sequencing of PIG13-DT. (B) 3’RACE sequencing of PIG13-DT. PIG13-DT was found to be 1160 nucleotides in length and contains a poly(A) tail.


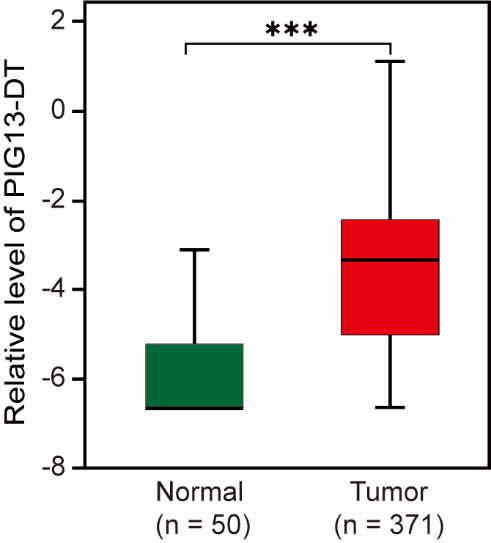


**Figure S3**. **Relative expression of PIG13-DT in HCC and normal tissues from the HCC TCGA database.** ****P* < 0.001.


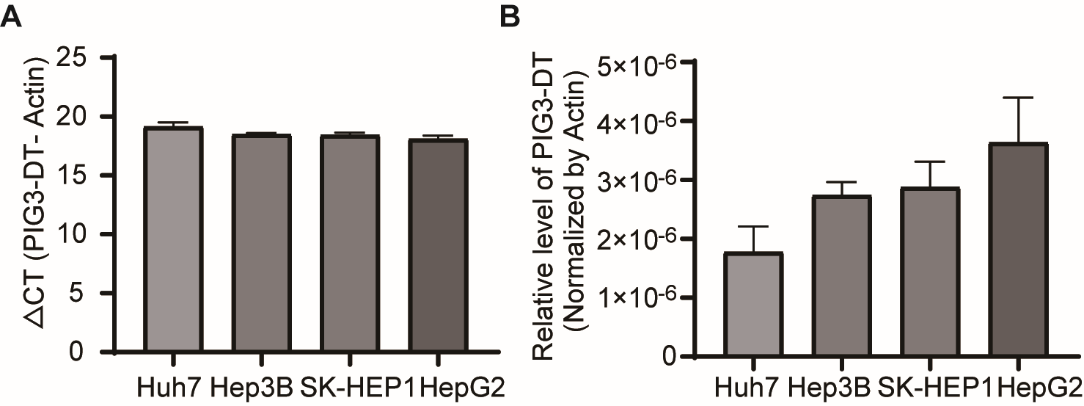


**Figure S4**. **Expression of PIG13-DT in HCC cells.** (A) Difference in cycle threshold (△CT) between PIG13-DT and Actin. (B) Relative expression of PIG13-DT normalized by Actin.


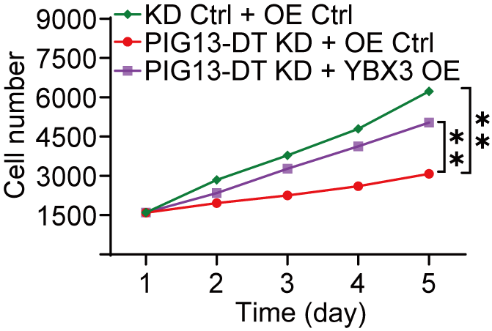


**Figure S5**. **The growth curve of Hep3B cell.** ***P* < 0.01.

**
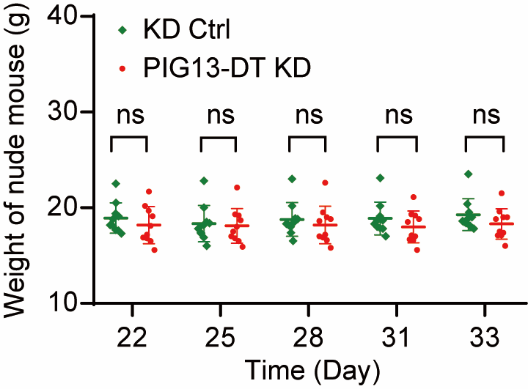
**

**Figure S6**. **Weight of nude mouse in the CDX model.** ns, *P* > 0.05.


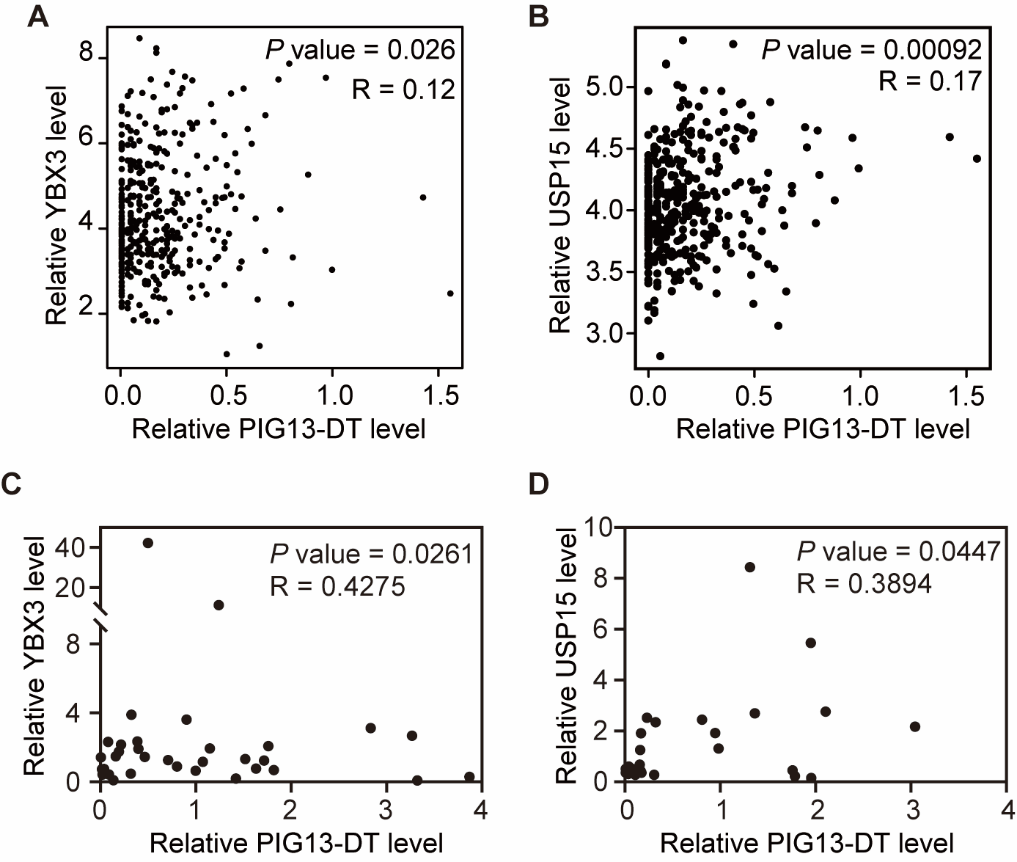


**Figure S7**. **Correlation between PIG13-DT and YBX3, as well as between PIG13-DT and USP15 in HCC patients.** (**A-D**) Positive correlation between PIG13-DT and YBX3, as well as PIG13-DT and USP15 in HCC samples from TCGA database (A, B) and local hospital (C, D).


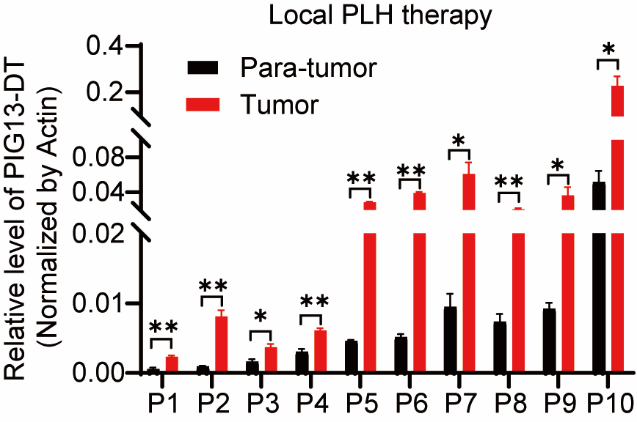


**Figure S8**. **Expression of PIG13-DT in HCC tissues**. Relative expression of PIG13-DT normalized by Actin. **P* < 0.05, ***P* < 0.01.
