# Supplementary material for: Unveiling the oncogenic role of lncRNA PIG13-DT in hepatocellular carcinoma progression
Source: Cancer Biol Ther. 2025 Oct 9;26(1):2567797. doi: 10.1080/15384047.2025.2567797 (PMC12520071; doi:10.1080/15384047.2025.2567797)
Supplement: Supplementary material — Supplementary Tables CLEAN COPY [file KCBT_A_2567797_SM8983.docx]

Table S1. **Pathological data of HCC patients with PLH therapy.**

| **Patient No.** | **Gender** | **Age** | **Grade** | **T tage** | **Metastasis** | **PVTT*** | **PFS**† | **OS**‡ | **PLH**§ |
| --- | --- | --- | --- | --- | --- | --- | --- | --- | --- |
| uHCC1 | male | 54 | C | IIIb | 0 | 0 | 177 | 324 | R |
| uHCC2 | male | 37 | b | IIb | 0 | 0 | 295 | 295 | R |
| uHCC3 | male | 50 | C | IIIb | 1 | 0 | 292 | 292 | R |
| uHCC4 | male | 73 | C | IIIb | 1 | III | 306 | 306 | R |
| uHCC5 | male | 67 | C | IIIa | 0 | II | 279 | 279 | R |
| uHCC6 | male | 49 | C | IIIa | 0 | II | 221 | 221 | R |
| uHCC7 | male | 54 | C | IIIa | 0 | I | 318 | 318 | R |
| uHCC8 | male | 53 | C | IIIb | 1 | III | 146 | 146 | nonR |
| uHCC9 | male | 47 | C | IIIb | 1 | III | 138 | 138 | nonR |
| uHCC10 | male | 55 | C | IIIa | 0 | I | 195 | 195 | nonR |
| uHCC11 | male | 54 | C | IIIa | 0 | III | 30 | 100 | nonR |
| uHCC12 | male | 46 | C | IIIa | 0 | III | 60 | 97 | nonR |
| uHCC13 | male | 55 | C | IIIb | 1 | I | 55 | 251 | nonR |
| uHCC14 | male | 57 | C | IIIa | 0 | I | 142 | 142 | nonR |

*PVTT: portal vein tumor thrombus; †PFS: progression free survival (day); ‡OS: overall survival (day); §PLH R: response, PLH nonR: non-response.

**Table S2. Full-length sequence of PIG13-DT.**

| **>lncRNA PIG13-DT, full length transcript wtih1160 nucleotides and poly-A tail**  GCGGAGGCCGCCGGGGCGCAGCGCGCTGGGGAGCGGGTTGGGGGCTGCTAGGGCTGCGGGGCGGGAAGGAGGCGGCAGTGTGTGTGTGTGTGTGTGTGTGTGTGTGTGTGTGTGTGTGTGTGTGGGCAGGTGTGTGTGTGTGTGTACGTGTGTGCGCGCGCGCCCTGCAGCGGCCGGCCAGCCGGAGGGAAGACCCCGGCGGGAAGACTGCGTCCCGTCCGGCCTTCCCGGGCCTGTCACTTGGGCGGTGGCGTGGGAGCGGGGAGGCTGCTCGGGGGGCAGCCGGGTGGGCCCAGCTCTAAGGGAGATTCGGGTCAGCCCCTGAGCCCAGCCTCCCGGCTGTGCCTGCTGGAAACAGACGGAGGGAAGGCCGGGCGTCTTTGGCCCACCGCGCGGCCGCTCCCCTCCCGGGCCAGGCTGAACGGGCGGGCGCAGCCCAGAGGCCCGGGCTCTGCCACCGATCCGCCTCCATACTGACCCGAGCTGCGGCGCCTGCAAGATTAATGAAGCATCCGCGATTCGGTAGAGGCGGCACGGGGAGGAAAGACACAAGATCGTGCTGCCCCCCTCTCCCCACCCTTCTCAGCCTCCTAGGACCTAAAGGCGTAGGCTGCGTCTGCTGGGCGCGTCCGGAGTGGTCCAGAGAGATCGCCATCCACCACTGCATCCGTCCAGTTCTTTGTGGACCCTCACAGAAGCTCAATCAATTTCCCACCCTCTGGAAAAGGGTATTTTTCTCTTCCCTGGCATATTGGGTGTTTCTTGTGTGCACCTAATTGATCTTTTTCCATTGATAGCCTTTGGATGTCTTTTGTGCGGCTTCCCCGCGCTTCACTGCTCCCTCATTGCCTTAGACACGCGAGGAATGTCAGGCCCCCAGAGTGAGAACACCCAAGAAAACAATAGGGGCAAAAGATCCTCCCTGGGCCTCGCGGATTATGCACAAACCAGATCATTTCCCACATTCGGCCAGGGTGAAGATGGAAAATGGAAAAGAACGCCATCAGAGTGGTGACACTGACGAGAAAAGCCAATTCATAATTTAGTTTAATGAAATGAGTTAGTAATCAACCACTAATGTCTAGGAAATTGGCCTTTTTTGAAAACTGGTTTTCCCTTTCTCCACCCCTTTTTTTTTCAAATTAAAAGACTAGTATCCCAAAAAAAAAAAAAAAA |
| --- |

**Table S3. Proteins pulled down by the PIG13-DT probe (identified by mass spectrometry).**

| **RBP protein** | **Sense MS*** | | **Anti-Sense MS** | |
| --- | --- | --- | --- | --- |
|  | **Peptides** | **PSMs**† | **Peptides** | **PSMs** |
| RBMXL1 | 10 | 11 | / | / |
| RPL36A | 5 | 8 | / | / |
| RBM17 | 5 | 5 | / | / |
| YBX3 | 2 | 4 | / | / |
| DAZAP1 | 3 | 4 | / | / |
| EIF2S2 | 27 | 41 | 1 | 1 |

*MS: mass spectrometric detection; †PSM: peptide-spectrum matches.

**Table S4. The sequence of USP15 targeted by YBX3 protein.**

| **>partial 3’Exon and 3’UTR sequence of USP15 from YBX3 eCLIPseq.** ATACTGCTTTTGCAAAAAATAAAGATGATGGAAAATGGTACTATTTTGATGACAGTAGTGTCTCCACTGCATCTGAAGACCAAATTGTGTCCAAAGCAGCATATGTACTCTTCTACCAGAGACAAGACACTTTCAGTGGAACTGGCTTTTTTCCTCTTGACCGAGAAACTAAAGGTGCTTCAGCTGCCACTGGCATCCCATTAGAAAGTGATGAAGATAGCAATGATAATGACAATGATATAGAAAATGAAAACTGTATGCACACTAACTAAtgaaagtcctagaagccataaaagagacactttcctgctggtggtatctatggaaatgatgaagttacccaccacattaaaacaaaagtctgagatggggagtttcagataaccgaatgtaaatcctttatcagattttaacttgtgcagtacttgaagtgaaacacaatgaaaactttaacagaaattgtctcttaatacatttacagtcttgtatttacaagctaaatatatataggaaatcacaAATAAAtcccttttaagtttg |
| --- |
